# A Minor Modification of Direct Browplasty Technique in a Patient with Brow Ptosis Secondary to Facial Paralysis: Copy-Paste-Excise and Stitch

**DOI:** 10.1155/2013/952079

**Published:** 2013-07-15

**Authors:** Arzu Taskiran Comez, Baran Gencer, Selcuk Kara, Hasan Ali Tufan

**Affiliations:** ^1^Department of Ophthalmology, School of Medicine, Canakkale Onsekiz Mart University, 17020 Canakkale, Turkey; ^2^Barbaros mahallesi, Plaj Sokak, Hamidiye Sitesi, C/9 Merkez, 17020 Canakkale, Turkey

## Abstract

*Purpose*. This report aimed to describe a minor modification of the traditional direct browplasty technique that aids in surgical planning for patients with brow ptosis secondary to facial paralysis without changing the shape of the brow. *Case Report*. A 74-year-old male patient with left facial paralysis secondary to chronic otitis media was referred with a complaint of low vision due to brow ptosis. We performed direct browplasty with a minor modification in order to aid a treatment customized to the patient. In this technique, a transparent film paper is used to copy the brow shape. A brow-shaped excision is facilitated just superior to the ptotic brow. *Conclusion*. The authors found that the *copy-paste-excise* and *stitch technique* was effective and successful for deciding the shape and the amount of excision that should be performed in patients with brow ptosis without resulting in asymmetrical, arched, and feminized brows.

## 1. Introduction

The brow consists of skin, subcutaneous tissue, muscle, and cilia and has a characteristic shape that is gender dependent [1]. In both men and women, the brow begins at the superomedial orbital rim and runs vertically through the medial canthus and nasal ala [2–4]. Although the beginning portion is similar, there are distinct differences between the two sexes. The normal position of the brow is just above the superior orbital rim in females and at the superior orbital rim in males. The female brow is more arched with a less prominent fat pad than males. In females, the brow arches above the supraorbital rim, whereas in men it arches only minimally along the rim [5].

The direct browplasty is suitable for any degree of brow ptosis secondary to injury of the temporal branch of the seventh cranial nerve in males as well as for older females with thick brows. It is an easy technique to perform, provides good control over the amount of brow lift, and gives predictable results [6]. The direct browplasty involves bilateral elliptical incisions immediately above the brows [7]. Closure of the elliptical excision area alters the brow's shape resulting in an arched brow with the maximum arch being at the center of the brow and feminized appearance [6–8]. Besides, arching may lead to a surprised look or “clown” appearance. With this report we aimed to prevent this over-lifted appearance and achieve symmetrically localized brows with a minor modification in traditional direct browplasty procedure in patients with brow ptosis.

## 2. Case Report

**Figure 1:**
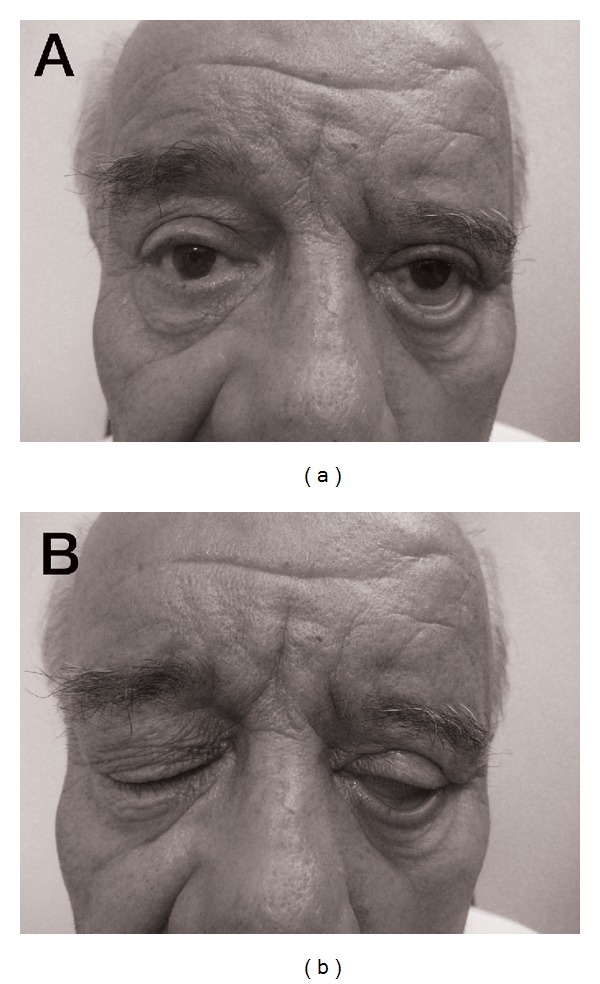
A 74-year-old man with left facial nerve palsy associated with marked brow ptosis, dermatochalasis, and lower eyelid laxicity (a). Complete closure of the right upper eyelid. Scleral show due to lower eyelid laxicity (b).

A 74-year-old Turkish male patient was referred with a complaint of low vision due to brow ptosis. He had facial paralysis secondary to chronic otitis media. Upon ophthalmological examination, the left brow was found to be localized 14 mm below its normal position with severe ectropion and horizontal laxicity of the lower eyelid resulting in scleral show (Figure 1(a)). The upper eyelid could be closed normally (Figure 1(b)).

The authors obtained written and oral permission from the patient for usage of his data. The formal consent signed by the patient of this study is available per request. 

## 3. The Copy-Paste-Excise and Stitch Technique

**Figure 2:**
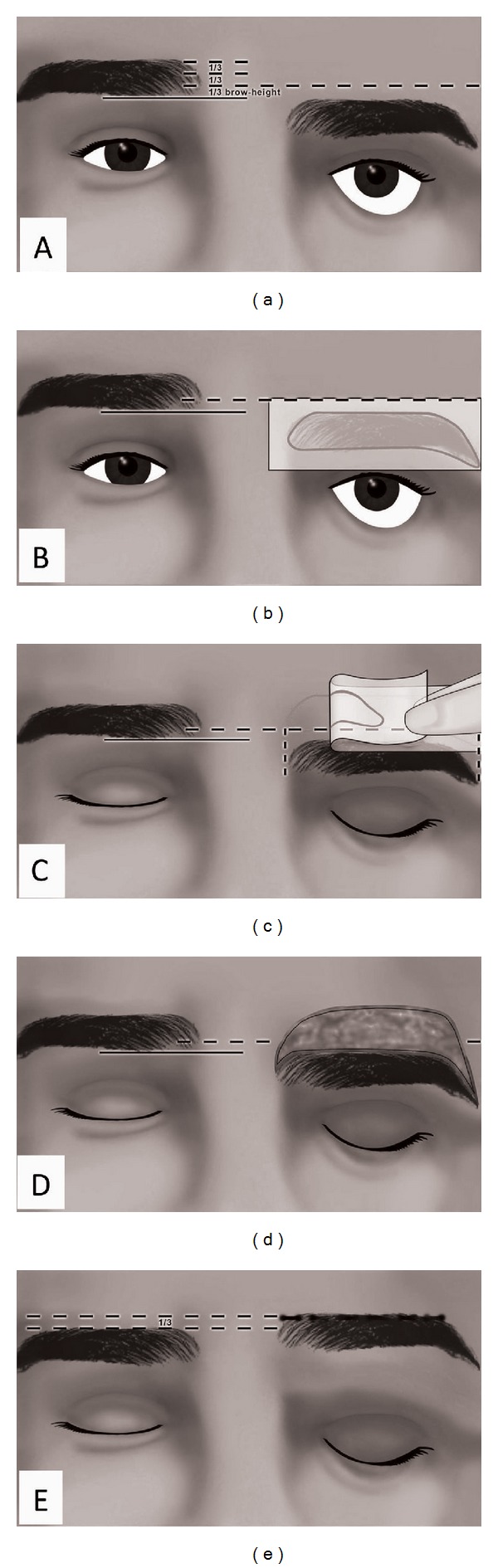
Interrupted line extends at the level of 1/3 height of normal brow to the ptotic brow. Continuous line demarcated the inferior edge of the contralateral brow (a). *Copy*. The brow shape is copied on a transparent paper (b). *Paste*. The brow copy is localized on the interrupted line. By pasting the paper, the brow shape is copied above the ptotic brow (c). *Excise*. Excision of the outlined area (d). *Stitch.* Direct and layered closure (e).

**Figure 3:**
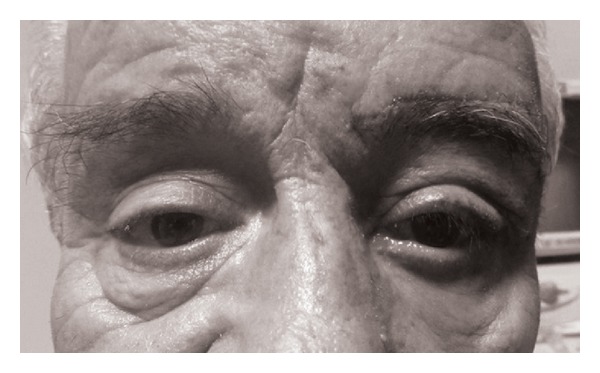
Postoperative 4-week view after brow lift with copy-paste-excise technique along with lateral canthal sling.

With the patient sitting upright, the normal brow is divided into 3 parts by height. A line is drawn starting from the inferior edge of the normal brow. The other (interrupted line) is drawn at inferior 1/3 part of the brow height. Since a lifted brow has a tendency to sag down to a lower position, an overcorrection of 1/3 of the normal brow height is attempted (Figure 2(a)). To achieve and warrant good symmetry for the brow position and shape, transparent paper is localized on the ptotic brow, and the shape of the brow is copied onto the paper (Figure 2(b)). The copied brow shape is also outlined in pen on the other side of the paper and is stuck just over the interrupted line that extends from the normal brow. The beginning and end points of the ptotic and copied brow are joined by vertical lines to complete the margins of the excision area (Figure 2(c)). All decisions were made with the patients' sitting and looking at the mirror. The first incision was performed along the contour of the brow at the upper row of hair. A brow-shaped excision was formed (Figure 2(d)). Since the patient had brow ptosis due to facial palsy, a periosteal fixation along with two-layered suturing was facilitated (Figure 2(e)). For horizontal laxicity, a lateral canthal sling was performed. The stiches were removed 10 days after the operation. During the 1st month of the follow-up period, the patient was happy and the result was satisfactory (Figure 3). 

## 4. Discussion

The direct browplasty is an easy technique to perform, provides good control over the amount of brow-lift, gives predictable results, and is suitable for any degree of brow ptosis. Booth and colleagues found that 32 of 43 cases of direct brow lift were satisfied or very satisfied based on the degree of lift obtained [6].

The main criticisms of this procedure are that it leaves a scar above the brow, forehead paresthesia is possible due to the damage of the supraorbital and supratrochlear nerves, and it alters the brow's shape resulting in an arched brow with the maximum arch being at the center of the brow [6–8]. The scar can be hidden in the brow hairs if performed in appropriate patients with thick brows. Paresthesias and numbness associated with damage to the supraorbital nerve are common complications of the procedure [7]. Transient decreases in sensation are often associated with divisions of smaller branches of the nerves, which typically resolve in several months [9]. Damage to the supraorbital nerve and vessels can be prevented by a dissection performed superficially to the frontalis muscle [7]. Booth and colleagues reported that 74% of patients experienced numbness and that 7% of patients were dissatisfied with this complication [6].

The direct browplasty involves elliptical incisions immediately above the brows [7]. The traditionally used technique for planning the height and the area of the ellipse/half-moon to be excised is as follows [6, 7, 10].

Mark the full length of the superior border of the brow adjacent to the uppermost brow hairs, pulling the brow up to its desired postoperative height [6, 7]. Repeat this maneuver at several sites while marking with a pen along the brow to outline the amount to be resected [6, 7]. Join these marks with a line to form an ellipse/half-moon. Excise this ellipse-shaped full-thickness skin incision down to the frontalis muscle [6, 7]. The width of the ellipse is measured to determine the desired elevation, and the length of the incision should extend along the entire brow [7]. An inferior dissection is performed below the superior orbital rims. Skin closure is completed in two levels with eversion of the wound. In the case of paralytic brow lift, overcorrection of the brow is recommended, as well as suturing to the periostium to prevent postoperative recurrence of ptosis [6, 7, 9, 10].

An excision of an ellipse superior to the brow actually lifts the brow; however, the greatest lift effect is achieved at the highest point of the ellipse that corresponds to the middle part of the brow. Although this shape may still suit some females, one may easily accept that a high and centrally arched brow is not suitable for men. It will also not work in women with unilateral brow ptosis with facial paralysis if the normal eye is not in an arch shape. Besides asymmetry and feminized appearance, this ellipse incision may lead to a surprised look, or “clown” appearance. Although this technique is mostly accepted as rehabilitative rather than cosmetic, any asymmetry, surprised look or clown-like appearance in the face may not be rehabilitative in either men or women. Another handicap of the traditional direct browplasty technique is that the width of the ellipse is determined subjectively as previously described [6, 7]. In this technique, we aim to lift the ptotic brow to a level that is 1/3 brow-thickness height above the normal brow's level. This point demarcates the superior border of the excision area. The area between the superior border down to the uppermost browline of the ptotic brow is the area to be excised. 

Since a male eyebrow is lower than the female brow and is straighter in contour, the surgical procedures to alter the position of the male eyebrow may actually feminize the patient's appearance if it is overlifted or if the arc of curvature is too exaggerated. The *copy-paste-excise and stitch* technique prevents this over-lifted appearance, gently locates the brow to its normal position, and reveals good symmetry. A careful dissection performed superficially to the frontalis muscle minimizes the damage to the supraorbital nerve and vessels. 

## 5. Conclusion

As a result, this simple technique demonstrates predictable, symmetrical, and satisfactory results. We have performed this procedure primarily in a male patient with facial paralysis; however, we think that it may be an option in both men and women with bilateral senile or unilateral brow ptosis who are pleased with the shape of their brows and who desires to bring the eyebrows in their proper position. A more quantitative measurement of outcomes and longer follow up with increasing number of patients including bilateral cases with senile brow ptosis are needed to determine the true objective efficacy of this technique.
